# Supplementary material for: Fusion protein EWS-FLI1 is incorporated into a protein granule in cells
Source: RNA. 2021 Aug;27(8):920–32. doi: 10.1261/rna.078827.121 (PMC8284321; doi:10.1261/rna.078827.121)
Supplement: Supplemental Material [file supp_27_8_920__DC1.html]

Fusion protein EWS-FLI1 is incorporated into a protein granule in cells — Fusion protein EWS-FLI1 is incorporated into a protein granule in cells — Supplemental Material 

# Fusion protein EWS-FLI1 is incorporated into a protein granule in cells

## Supplemental Material

- Supplemental\_Table\_1.xlsx
- Supplemental\_Table\_2.xlsx
- Supplemental\_Table\_3.xlsx
- Suppplemental\_Figures.pptx
