# Supplementary material for: Fusion protein EWS-FLI1 is incorporated into a protein granule in cells
Source: RNA. 2021 Aug;27(8):920–32. doi: 10.1261/rna.078827.121 (PMC8284321; doi:10.1261/rna.078827.121)
Supplement: Supplemental Material [file supp_078827.121_Suppplemental_Figures.pptx]

## Slide 1
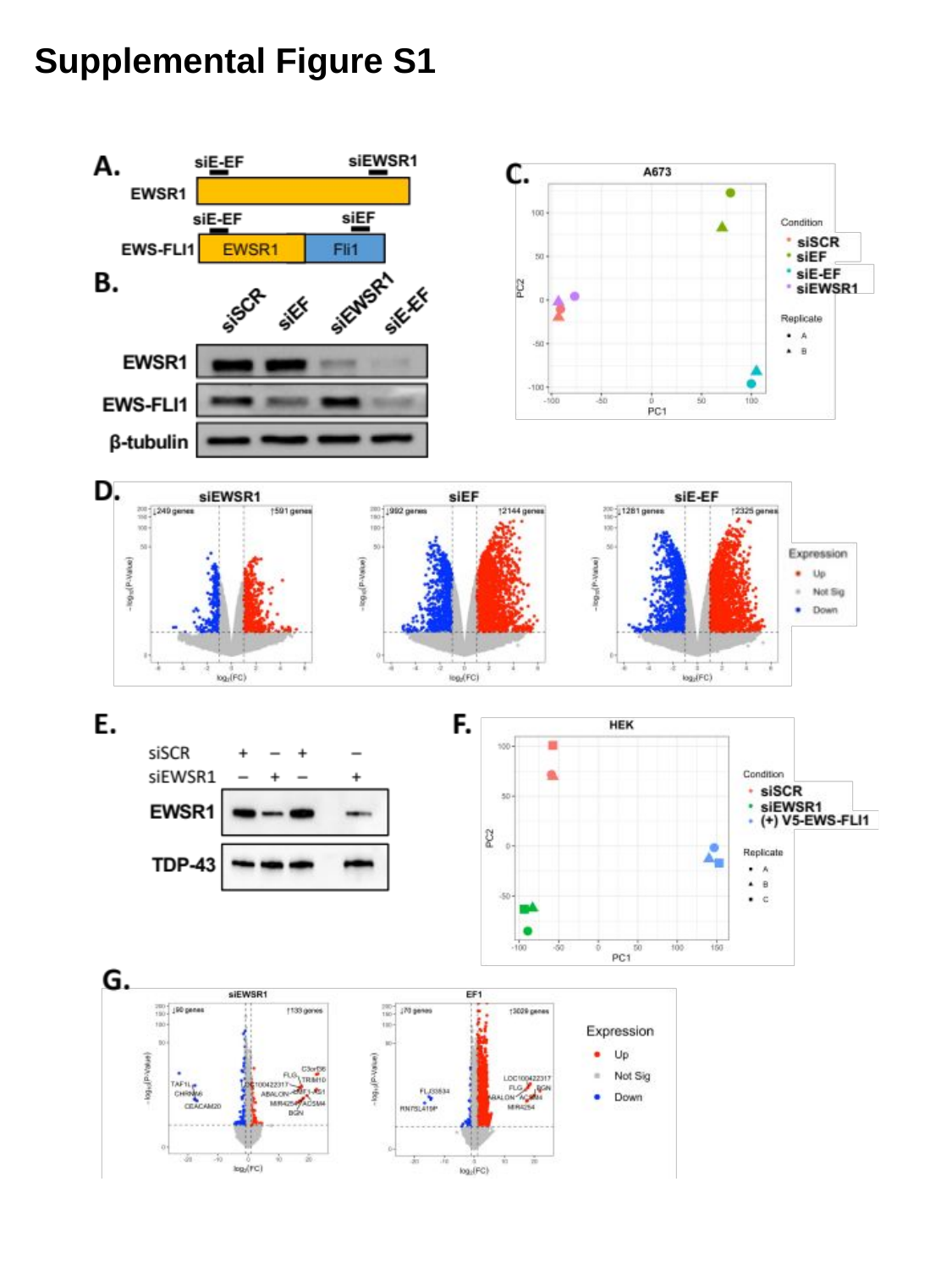

# Supplemental Figure S1

## Slide 2
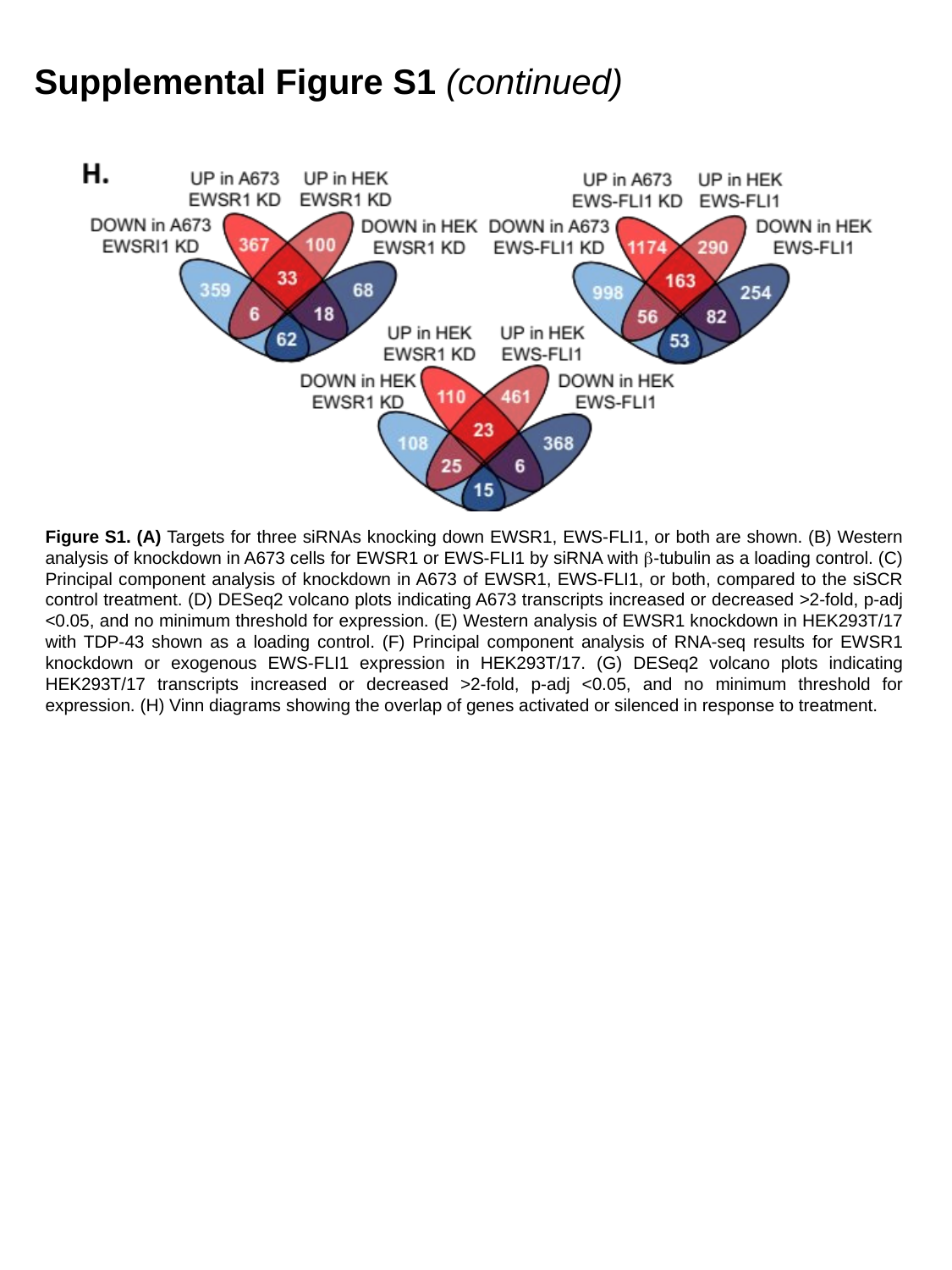

# Supplemental Figure S1 (continued)
Figure S1. (A) Targets for three siRNAs knocking down EWSR1, EWS-FLI1, or both are shown. (B) Western analysis of knockdown in A673 cells for EWSR1 or EWS-FLI1 by siRNA with b-tubulin as a loading control. (C) Principal component analysis of knockdown in A673 of EWSR1, EWS-FLI1, or both, compared to the siSCR control treatment. (D) DESeq2 volcano plots indicating A673 transcripts increased or decreased >2-fold, p-adj <0.05, and no minimum threshold for expression. (E) Western analysis of EWSR1 knockdown in HEK293T/17 with TDP-43 shown as a loading control. (F) Principal component analysis of RNA-seq results for EWSR1 knockdown or exogenous EWS-FLI1 expression in HEK293T/17. (G) DESeq2 volcano plots indicating HEK293T/17 transcripts increased or decreased >2-fold, p-adj <0.05, and no minimum threshold for expression. (H) Vinn diagrams showing the overlap of genes activated or silenced in response to treatment.

## Slide 3
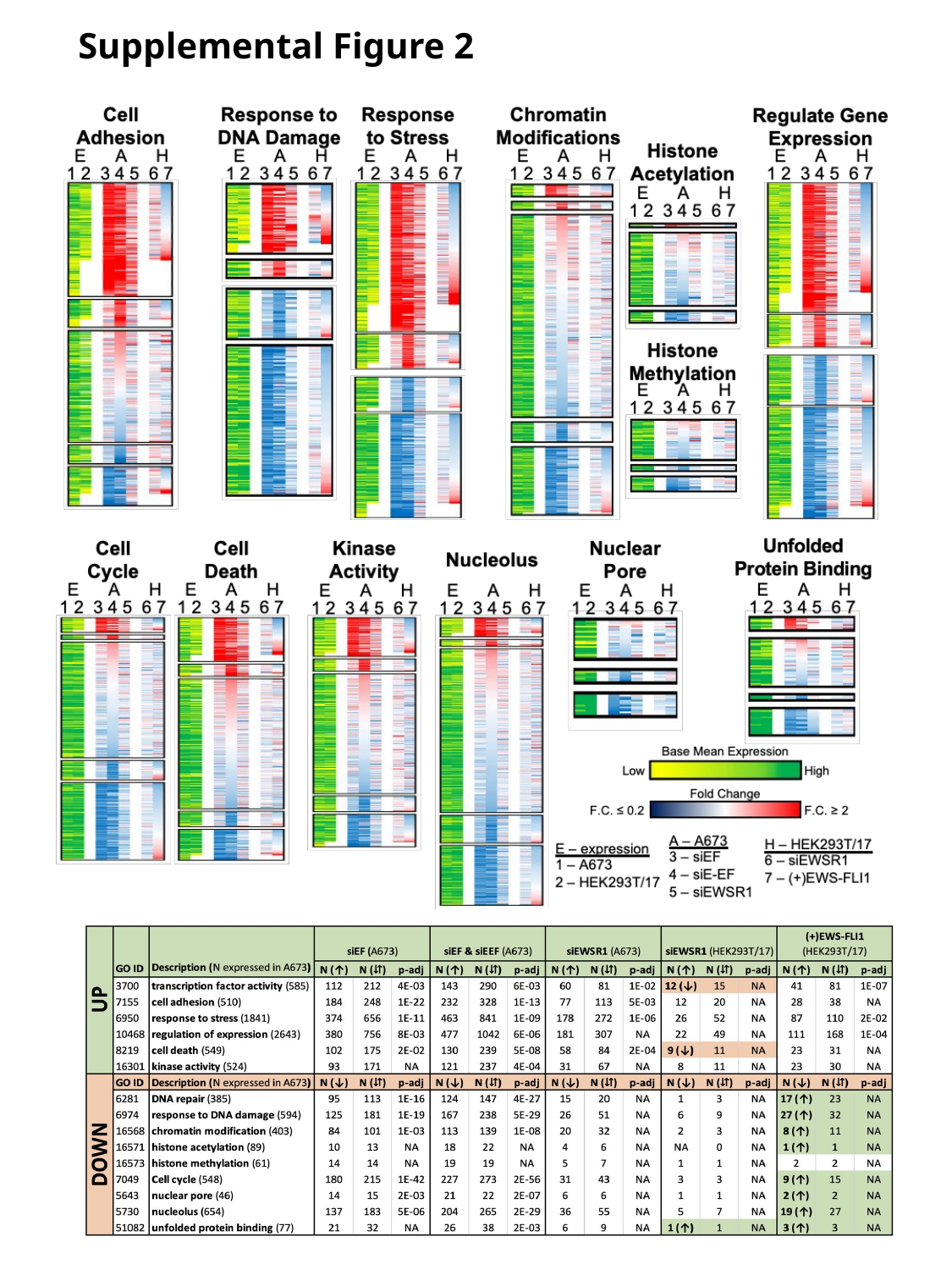

# Supplemental Figure 2

## Slide 4
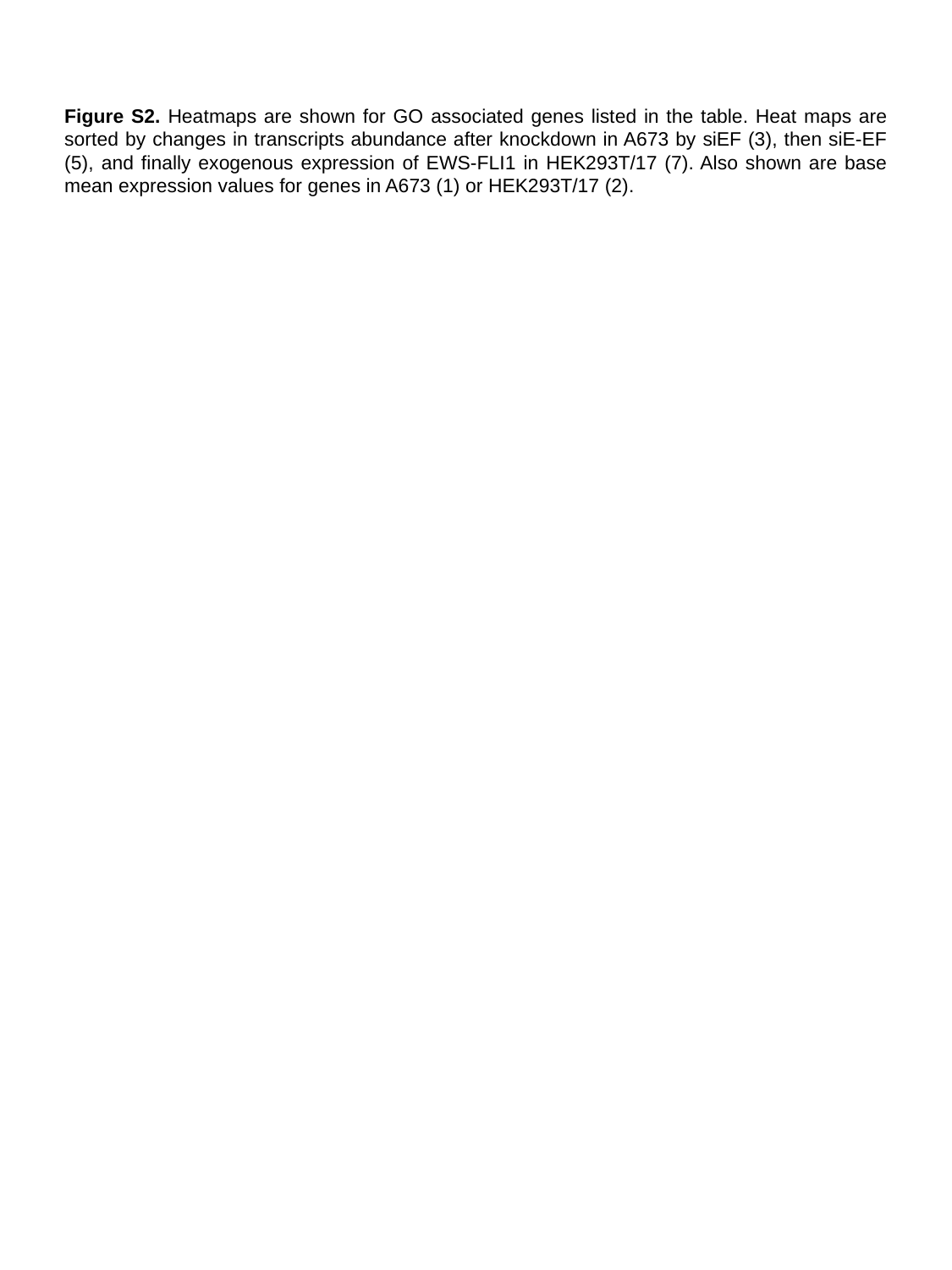

Figure S2. Heatmaps are shown for GO associated genes listed in the table. Heat maps are sorted by changes in transcripts abundance after knockdown in A673 by siEF (3), then siE-EF (5), and finally exogenous expression of EWS-FLI1 in HEK293T/17 (7). Also shown are base mean expression values for genes in A673 (1) or HEK293T/17 (2).

## Slide 5
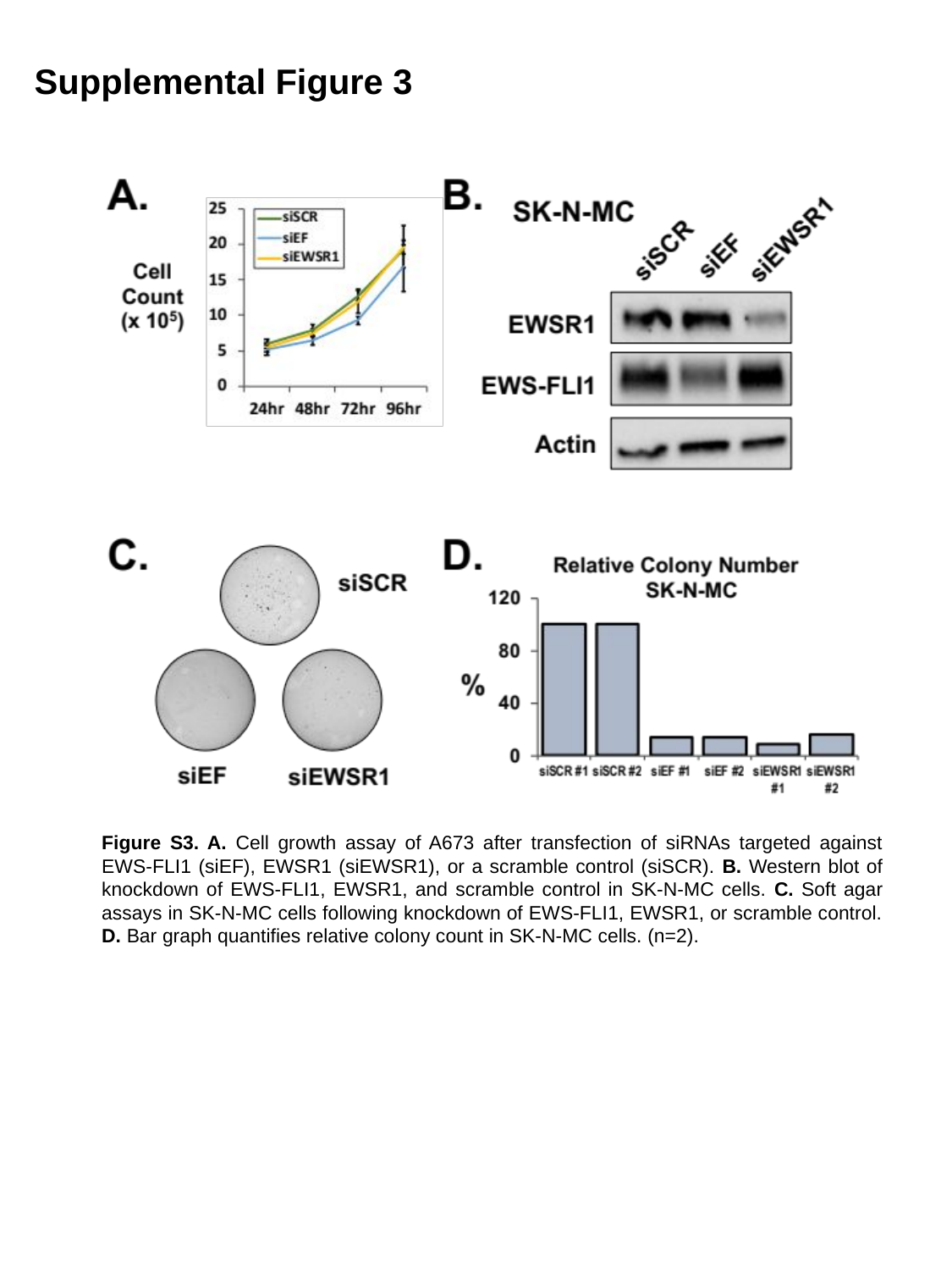

# Supplemental Figure 3
Figure S3. A. Cell growth assay of A673 after transfection of siRNAs targeted against EWS-FLI1 (siEF), EWSR1 (siEWSR1), or a scramble control (siSCR). B. Western blot of knockdown of EWS-FLI1, EWSR1, and scramble control in SK-N-MC cells. C. Soft agar assays in SK-N-MC cells following knockdown of EWS-FLI1, EWSR1, or scramble control. D. Bar graph quantifies relative colony count in SK-N-MC cells. (n=2).

## Slide 6
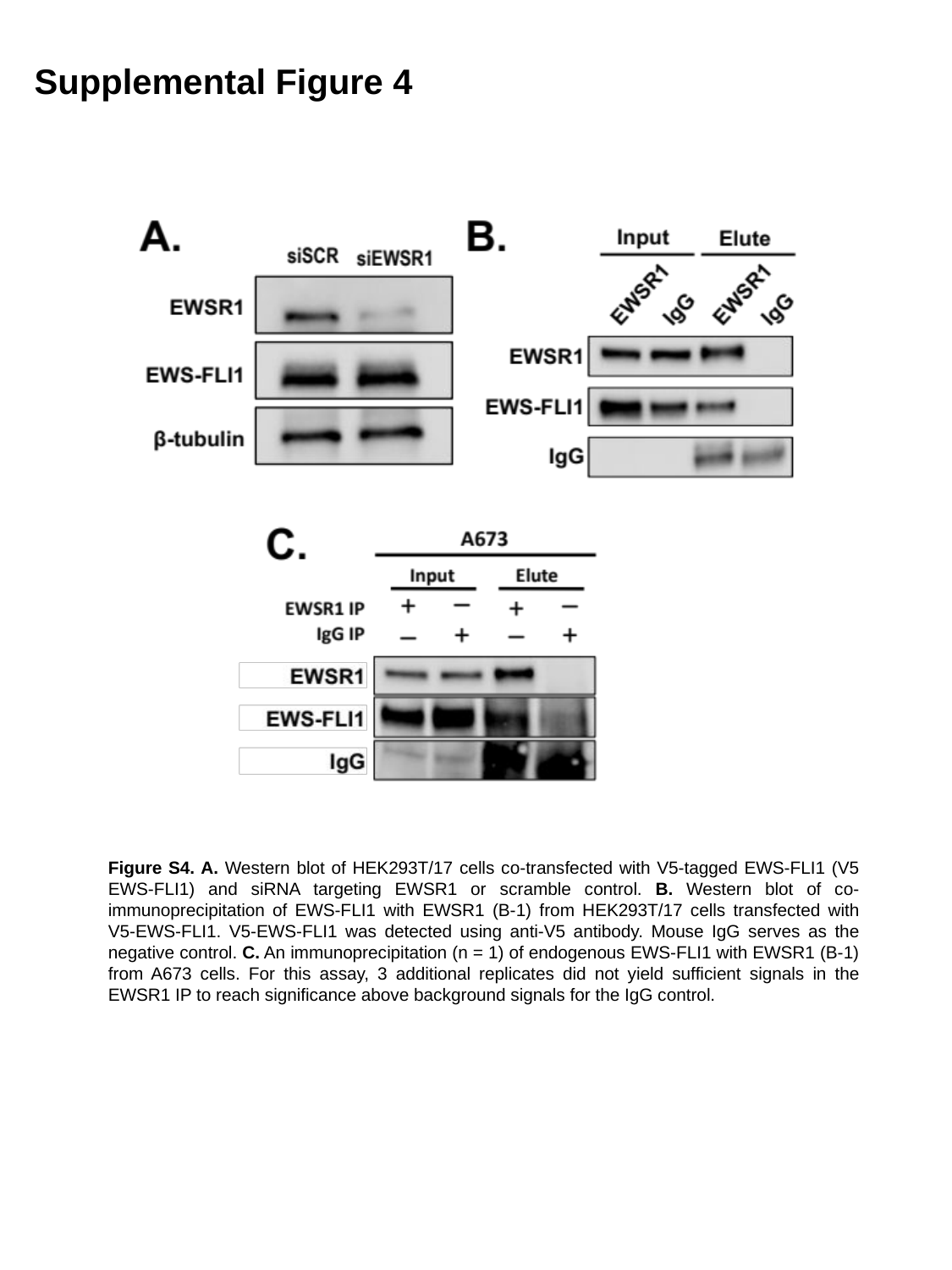

# Supplemental Figure 4
Figure S4. A. Western blot of HEK293T/17 cells co-transfected with V5-tagged EWS-FLI1 (V5 EWS-FLI1) and siRNA targeting EWSR1 or scramble control. B. Western blot of co-immunoprecipitation of EWS-FLI1 with EWSR1 (B-1) from HEK293T/17 cells transfected with V5-EWS-FLI1. V5-EWS-FLI1 was detected using anti-V5 antibody. Mouse IgG serves as the negative control. C. An immunoprecipitation (n = 1) of endogenous EWS-FLI1 with EWSR1 (B-1) from A673 cells. For this assay, 3 additional replicates did not yield sufficient signals in the EWSR1 IP to reach significance above background signals for the IgG control.

## Slide 7
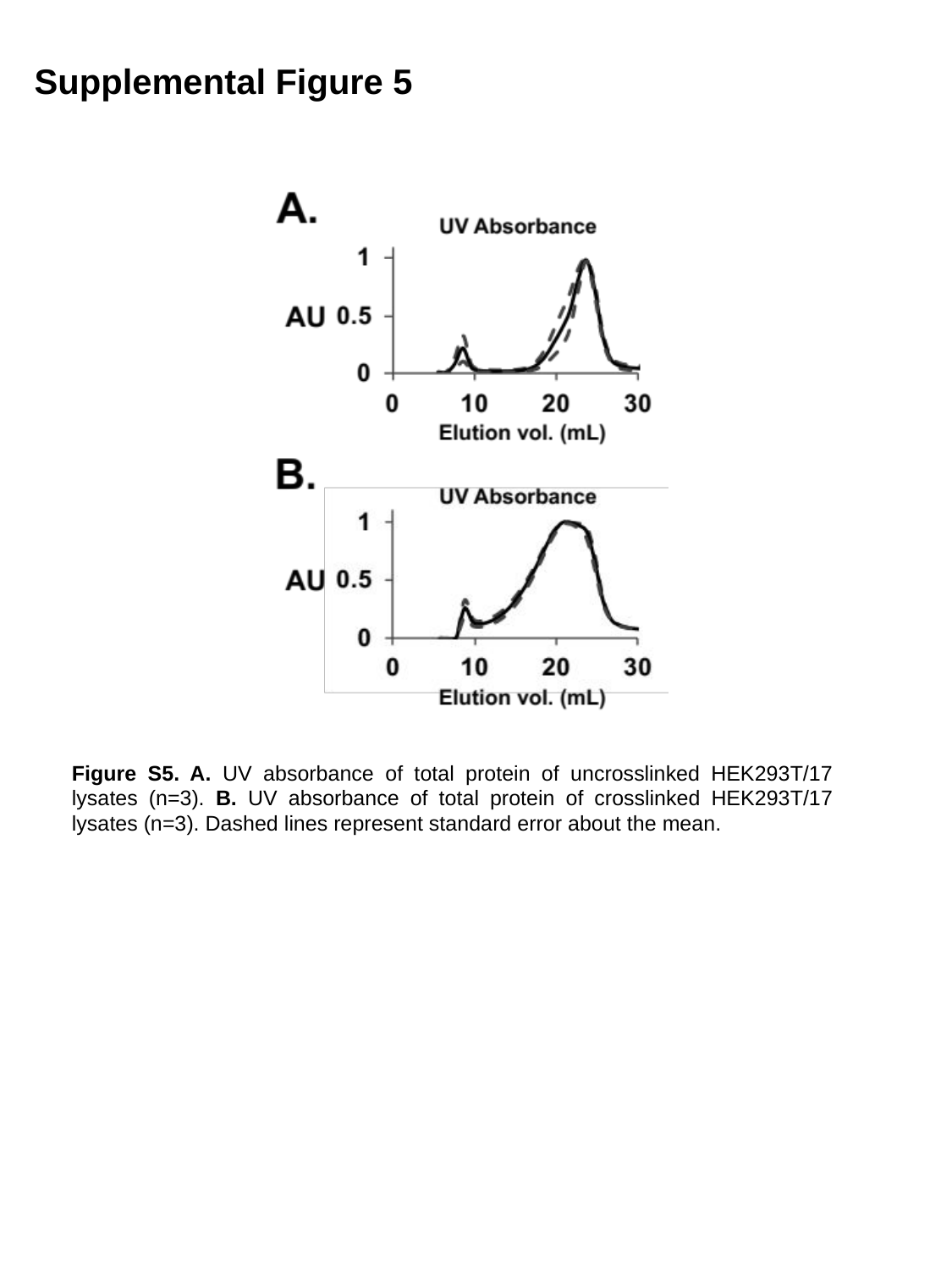

# Supplemental Figure 5
Figure S5. A. UV absorbance of total protein of uncrosslinked HEK293T/17 lysates (n=3). B. UV absorbance of total protein of crosslinked HEK293T/17 lysates (n=3). Dashed lines represent standard error about the mean.

## Slide 8
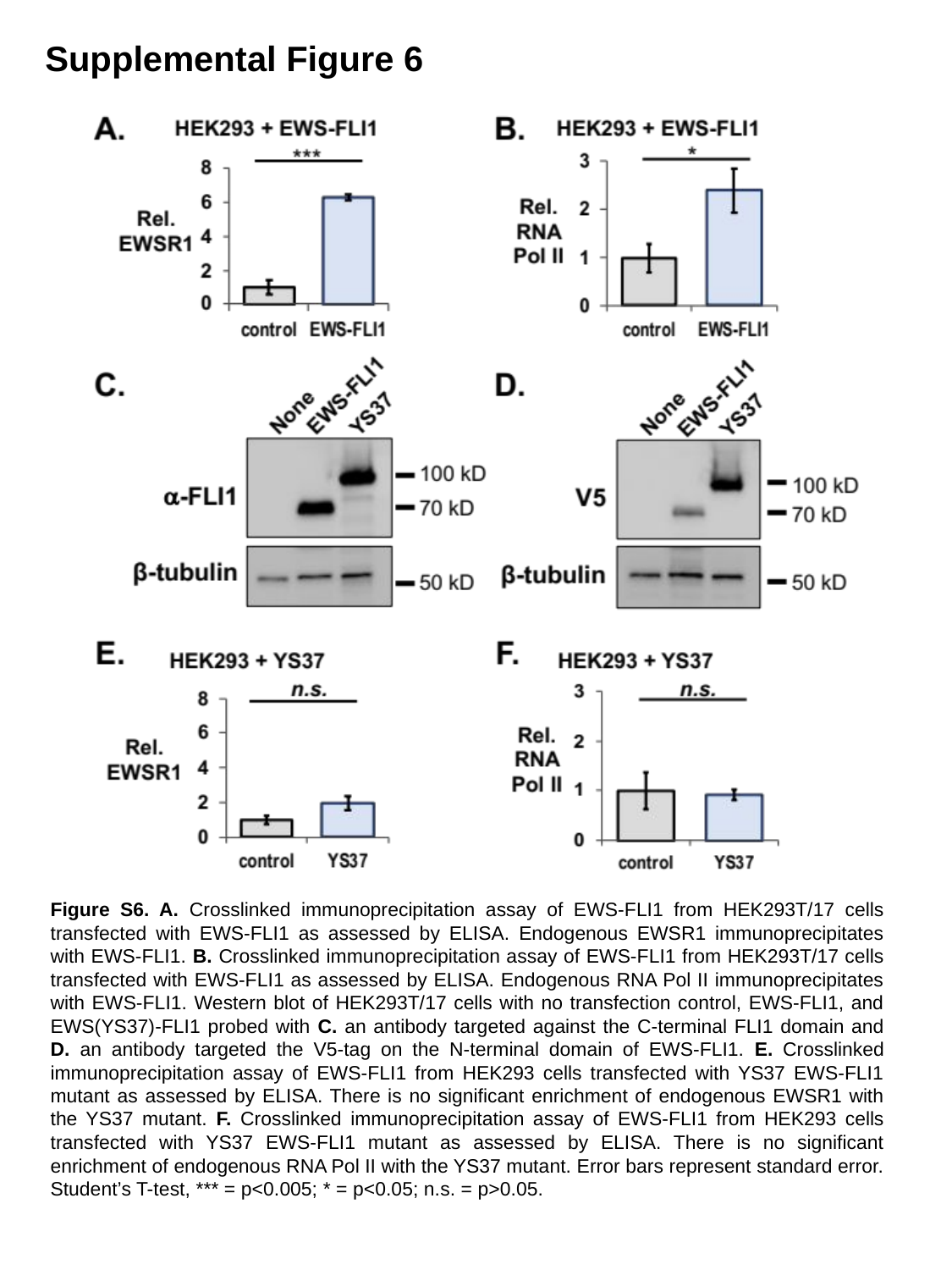

Supplemental Figure 6
Figure S6. A. Crosslinked immunoprecipitation assay of EWS-FLI1 from HEK293T/17 cells transfected with EWS-FLI1 as assessed by ELISA. Endogenous EWSR1 immunoprecipitates with EWS-FLI1. B. Crosslinked immunoprecipitation assay of EWS-FLI1 from HEK293T/17 cells transfected with EWS-FLI1 as assessed by ELISA. Endogenous RNA Pol II immunoprecipitates with EWS-FLI1. Western blot of HEK293T/17 cells with no transfection control, EWS-FLI1, and EWS(YS37)-FLI1 probed with C. an antibody targeted against the C-terminal FLI1 domain and D. an antibody targeted the V5-tag on the N-terminal domain of EWS-FLI1. E. Crosslinked immunoprecipitation assay of EWS-FLI1 from HEK293 cells transfected with YS37 EWS-FLI1 mutant as assessed by ELISA. There is no significant enrichment of endogenous EWSR1 with the YS37 mutant. F. Crosslinked immunoprecipitation assay of EWS-FLI1 from HEK293 cells transfected with YS37 EWS-FLI1 mutant as assessed by ELISA. There is no significant enrichment of endogenous RNA Pol II with the YS37 mutant. Error bars represent standard error. Student’s T-test, *** = p<0.005; * = p<0.05; n.s. = p>0.05.

## Slide 9
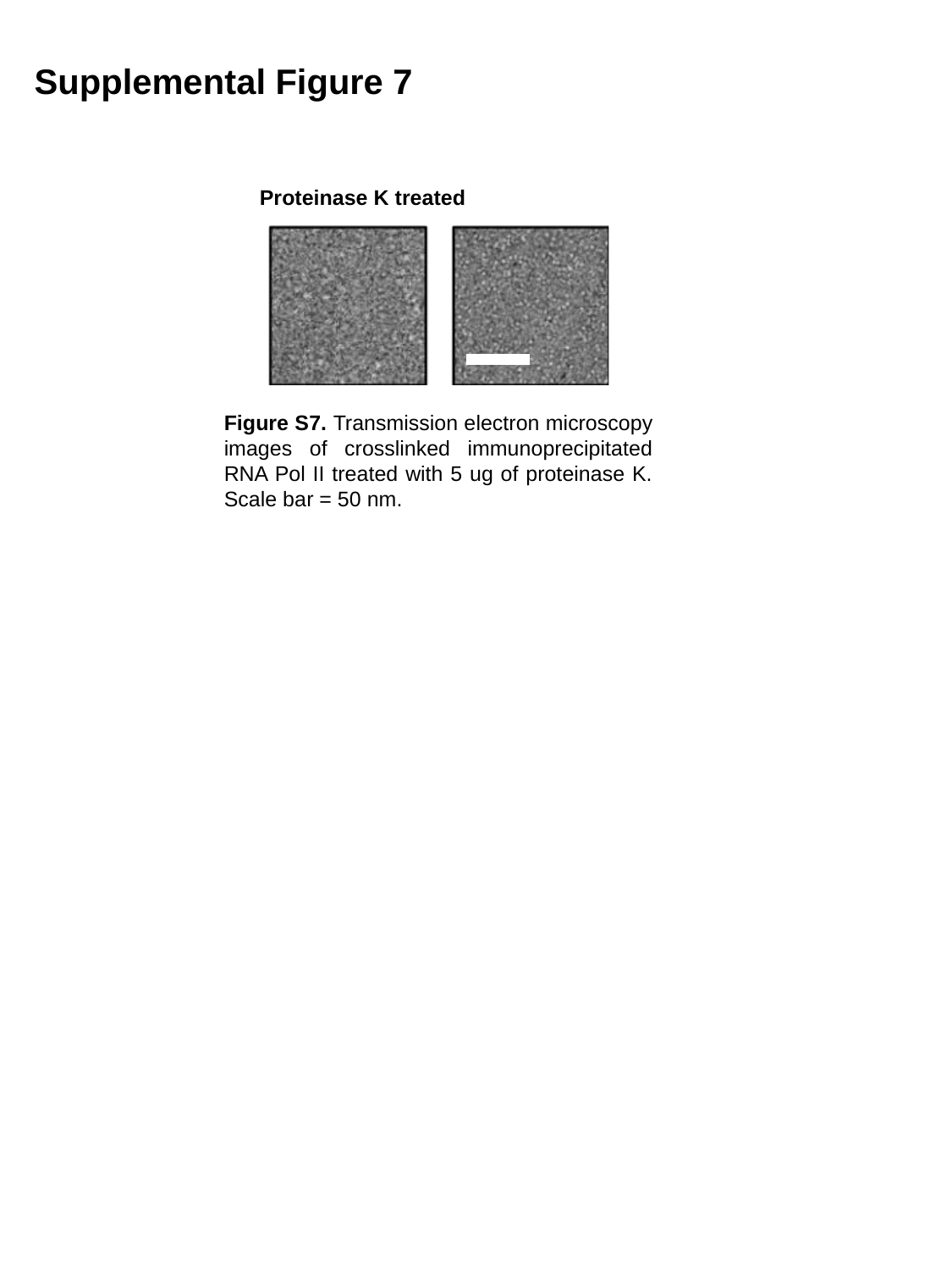

# Supplemental Figure 7
Proteinase K treated
Figure S7. Transmission electron microscopy images of crosslinked immunoprecipitated RNA Pol II treated with 5 ug of proteinase K. Scale bar = 50 nm.
